# Peripheral intravascular catheter-associated bloodstream infection in the medical–surgical ICU

**DOI:** 10.1017/ash.2022.111

**Published:** 2022-05-16

**Authors:** Nancy Hogle, Patrick Burke, Thomas Fraser

## Abstract

**Background:** Prompt removal of unnecessary central venous catheters (CVC) may reduce central-line–associated bloodstream infection (CLABSI). Primary non–central-line–associated hospital-acquired bloodstream infection (BSI), including peripheral intravascular (PIV) catheter-associated bloodstream infection (PIVABSI) remains a problem. Hospitals use CLABSI surveillance data to measure patient safety, yet this measure alone fails to describe the burden of total intravascular device-related infection. We described non-CLABSI primary BSI due to PIV in our medical–surgical ICU population. **Methods:** Hospital-wide surveillance for primary hospital-acquired BSI, including CLABSI, was conducted in accordance with NHSN protocol. We measured PIV catheter days and central-line days using a database including nursing device documentation and patient census data to count the number of patients with 1 or more devices in place in each location, counted at the same time each day. By substituting the role of the CVC with short or midline PIV in NHSN CLABSI surveillance protocols, we performed surveillance for PIVABSI. We defined PIVABSI as a patient without CVC and either a short or midline catheter in place for >2 calendar days on the date of BSI. Patients with BSI and both CVC and PIV were counted as CLABSI. We compared CVC and PIV utilization and the incidence density of CLABSI and PIVABSI in 8 medical and surgical ICUs at our large teaching hospital. We used OpenEpi version 3.01 software to test the hypothesis that the incidence density of CLABSI would be significantly different from that of PIVABSI. **Results:** From January to September 2021, there were 16 CLABSIs and 12 primary non–central-line–associated hospital-acquired BSIs, all 12 were PIVABSIs. Of these 12, 8 had >1 PIV in place and none were midlines. There were 13,418 central-line days, 10,897 short and midline peripheral IV days, and 22,415 patient days, resulting in device utilization ratios of 0.60 and 0.49, respectively. The incidence density of CLABSI was 1.2 per 1,000 central-line days, although the incidence density of PIVABSI was 1.1 per 1,000 peripheral IV days (*P* = .84). There was no difference in pathogens between the 2 groups. **Conclusions:** PIVABSI represented more than one-third of the total primary hospital-acquired BSIs in our medical and surgical ICUs. Total BSI surveillance is feasible. Efforts to reduce CLABSI should be part of a broader strategy to decrease total hospital-acquired BSI from all vascular access devices.

**Funding:** None

**Disclosures:** None

